# Examining the Factor Structure and Subgroup Invariance of the Deliberate Denial of Disordered Eating Behaviors Scale

**DOI:** 10.3390/bs16060898

**Published:** 2026-06-02

**Authors:** Lindsay Howard, Sage Hawn, Kayla D. Pitchford, Riley McGrath, Aubri Farniok, Arturo Sesma, Kristin E. Heron

**Affiliations:** 1 Department of Psychology, Augustana University, 2001 S. Summit Ave., Sioux Falls, SD 57197, USA; 2 The Virginia Consortium Program in Clinical Psychology, 234 Mills Godwin Life Sciences Building, Norfolk, VA 23529, USA; shawn@odu.edu (S.H.); kpitc001@odu.edu (K.D.P.); kheron@odu.edu (K.E.H.); 3 Department of Psychology, Old Dominion University, 1320 44th St. Mills Godwin Life Sciences Building (MGB), Norfolk, VA 23508, USA; 4 Department of Psychology, St. Catherine University, 2004 Randolph Avenue, St. Paul, MN 55105, USAagsesma@stkate.edu (A.S.)

**Keywords:** denial, disordered eating, eating disorders, factor analysis, measurement

## Abstract

The denial, concealment, or omission of disordered eating behaviors has received limited attention in the eating disorder literature, partly due to the lack of reliable and valid measures. This study re-examined the factor structure of the Denial of Disordered Eating Behaviors Scale (DDEBS-12) and tested measurement invariance across gender identity, racial identity, and levels of disordered eating in a non-clinical undergraduate sample (*N* = 3285). Confirmatory factor analyses did not support the original unidimensional structure. Instead, a seven-item model assessing denial of dietary restriction demonstrated the best fit and was renamed the DDEBS-restriction. The revised scale showed good internal consistency and demonstrated expected associations with measures of concealment, disclosure, and dietary restriction, supporting convergent and criterion validity. Measurement invariance analyses supported configural and metric invariance across men and women, Black and White participants, and individuals with non-clinical versus elevated levels of disordered eating; scalar invariance was not supported across disordered eating severity groups. These findings suggest that the DDEBS-restriction demonstrates promising psychometric properties within an undergraduate sample, though additional research is needed to establish its generalizability across broader and more diverse populations.

## 1. Introduction

Disordered eating includes behaviors such as chronic dieting, irregular eating patterns, and the use of unhealthy weight control methods that pose a variety of risks, including problems with nutritional deficiencies, comorbid psychopathology, and impaired relationships (Alsheweir et al., 2023). One characteristic of disordered eating that has received relatively little attention is the denial of disordered eating, or the tendency to conceal engagement in disordered eating behaviors (Howard et al., 2020; Schoen et al., 2012; Vandereycken & Van Humbeeck, 2008). Denial of disordered eating can be defined as any conscious omission, concealment, or misrepresentation of behavior related to disordered eating (Howard et al., 2020). Efforts to misrepresent or shield one’s eating behaviors are recurrent characteristics among individuals with eating disorders (Schoen et al., 2012; Vandereycken & Van Humbeeck, 2008), and these evasive behaviors may contribute to the persistence and severity of these conditions. Individuals with disordered eating conceal symptoms for several reasons. Some view their behaviors as part of their identity (egosyntonicity) and “fake good” to protect their sense of self (Vandereycken, 2006). Others hide behaviors because they provide a sense of achievement or control and fear intervention (Vitousek et al., 1998). Most conceal symptoms to avoid being labeled and stigmatized (Vandereycken, 2006). Importantly, denial of disordered eating may emerge within specific developmental and interpersonal contexts. During adolescence and the transition to emerging adulthood, increasing peer influence and stigma surrounding eating and body image may increase the use of concealment and secrecy around eating behaviors (K. L. Rose et al., 2022). Therefore, denial may reflect not only current symptom severity but also developmentally rooted patterns of interpersonal regulation and self-presentation. Stigma involved with disordered eating, as well as related denial of engagement in disordered eating, are positively related to eating disorder symptomatology (Griffiths et al., 2018) and may be a particularly salient characteristic of individuals ultimately diagnosed with anorexia nervosa (Abbate-Daga et al., 2013; Vitousek et al., 1998). Thus, assessment tools that identify these behaviors are essential for developing effective prevention and intervention efforts for individuals across the spectrum of eating behavior disturbances.

To address this lacuna in assessing eating secrecy, Howard et al. (2020) developed the Deliberate Denial of Disordered Eating Behaviors Scale (DDEBS-12) to measure the extent and manner in which individuals consciously deny their disordered eating behaviors. The DDEBS-12 aimed to capture how individuals may misrepresent their food consumption using a 12-item scale (e.g., “How often have you told people you have eaten when you have not?”). Although related to broader constructs such as social desirability, impression management, and self-concealment, denial of disordered eating reflects a more behavior-specific process. The scale was ultimately designed to assess denial of disordered eating by asking questions about denial of eating habits (e.g., hiding food, being dishonest about eating, etc.) that could be answered by anyone, regardless of eating disorder symptomatology. The DDEBS-12 was not designed to assess specific eating behaviors (e.g., denial of binge eating, loss of control over eating) to avoid conflating denial of specific behaviors versus a lack of engagement in the behavior itself.

The psychometric properties of the DDEBS-12 were initially assessed with two samples of college women (total *N* = 673) who self-reported subclinical or clinical levels of disordered eating using EDE-Q cutoffs. Using exploratory and confirmatory factor analyses across these two samples, a unidimensional factor structure was reported (Howard et al., 2020). However, further research is needed because the initial study lacked the power for subgroup analyses. The DDEBS-12 was developed with a sample of college women who reported, at least, subclinical disordered eating behavior. Thus, one purpose of this paper is to explore whether the factor structure of the DDEBS-12 replicates within a non-clinical sample and to test for invariance of the items across levels of engagement in disordered eating (i.e., non-clinical vs. subclinical or above, as defined by cut-off scores from the Eating Disorder Examination Questionnaire; Fairburn & Beglin, 1994; Mond et al., 2004; Schaefer et al., 2018).

Another focus of the current study is to examine the factor structure of the DDEBS-12 across racial and gender identity. The original DDEBS-12 study used a female-identified-only sample because eating disorders are more prevalent among women than men. However, recent research suggests that subclinical disordered eating may be just as common in men as in women (Mitchison et al., 2019; Trompeter et al., 2021). Any assessment of disordered eating, therefore, should examine if men and women respond similarly. Lastly, while non-majority women comprised the initial studies’ samples, all women were analyzed together, and it has yet to be tested whether the unidimensional factor structure holds across different racial identity groups. Halbeisen et al. (2022) implore researchers to incorporate more diverse samples in their studies of disordered eating, as research in the field of disordered eating has consisted mostly of White women.

Thus, the purpose of the present study is threefold. The first goal is to re-examine the factor structure of the DDEBS-12 in a broader sample and conduct alternative model testing based on theoretical rationale should the original factor structure not hold. The second goal is to determine whether the factor structure and loadings hold across different racial and gender identity groups and levels of disordered eating (non-clinical vs. self-reported subclinical or clinical). The last goal is to re-assess the reliability and validity of the DDEBS in an independent sample. First, it was hypothesized that configural (equality of the factor structure) and metric (equality of the factor loadings) tests would be invariant across these various groups based on the assumption that denial of disordered eating reflects a similar underlying construct across populations; however, it was expected that there would be variance at the scalar (equality of the intercepts) level, such that women and individuals who engage in, at least, subclinical levels would report higher DDEBS means than men and non-clinical participants. Scalar differences based on racial identity were exploratory. Second, it was hypothesized that the scale would show, at least, acceptable internal consistency as well as demonstrate criterion and convergent validity, as assessed by correlations with measures of disordered eating, body dissatisfaction, concealment, and disclosure.

## 2. Materials and Methods

### 2.1. Procedure

Data for the current paper were drawn from a large southeastern public university in North America. The study exclusively recruited participants through the university’s psychology participant pool from 2018 to 2022. Participants were informed that the purpose of the study was to collect information about college students’ health, well-being, and general life experiences and attitudes. In exchange for course credit, participants completed a series of online questionnaires. All participants were required to be at least 18 years old and provide informed consent. The study was approved by the institution’s research ethics review board.

### 2.2. Participants

As part of a larger study about college health and experiences, the participant sample consisted of 4517 students. Of these, 553 were removed because they were duplicate entries, did not correctly answer at least 3 out of 4 attention items, or completed the survey in less than one-third of the median duration time. Additionally, prior to running analyses, individuals who scored 1 SD above the mean (17%; *n* = 679) on a measure designed to detect individuals unable to accurately report on their eating behaviors were removed from subsequent analyses^1^. This measure was utilized given that the researchers asked participants to report on behaviors that they typically deny (see Underreporting of Disordered Eating Behaviors Scale in measures section for further information). Thus, a total of 3285 students (77.2% Women, 22.8% Men) were included in the current study with a mean age of 21.28 (*SD* = 6.79) and similar numbers of White (55.0%) and Black (45.0%) participants. Analyses were limited to participants identifying as Black or White, and individuals identifying with other racial or ethnic groups were excluded due to sample size constraints. Participants were also subdivided by various levels of engagement in disordered eating (63.0% non-clinical, 37% subclinical or above), as determined by cutoffs of 1.68 for men and 2.3 for women on the Eating Disorder Examination Questionnaire (EDE-Q; Mond et al., 2004; Schaefer et al., 2018). Table 1 provides demographic information for the participant sample.

### 2.3. Measures

#### 2.3.1. Demographics

A set of demographic questions was administered asking participants to self-report their age, racial identity, gender identity, sexual orientation, and student classification status.

#### 2.3.2. Denial of Disordered Eating

The Deliberate Denial of Disordered Eating Behaviors (DDEBS-12; Howard et al., 2020) is a 12-item self-report measure that assesses the extent to which individuals who engage in disordered eating lie about such behaviors (e.g., “How often have you deliberately hid food (e.g., in a napkin) in order to give the impression you ate more than you did?”) in the last month. Item responses are scored on a 7-point Likert scale ranging from 1 (never) to 7 (every time). Total scores are calculated by summing all items, with higher scores demonstrating more denial of disordered eating behaviors. The DDEBS-12 exhibits excellent internal consistency (α = 0.92) and was validated in a sample of college women with, at least, subclinical levels of eating pathology (Howard et al., 2020). The DDEBS-12 total score is positively correlated with self-concealment (r = 0.30) and negatively correlated with distress disclosure (r = −0.28), demonstrating convergent validity. Internal consistency for the present sample was excellent, with Cronbach’s alpha of 0.93 and McDonald’s omega of 0.95.

#### 2.3.3. Disordered Eating

The Eating Disorder Examination Questionnaire (EDE-Q; Fairburn & Beglin, 1994) is a 29-item self-report measure that assesses eating disorder pathology in the last 28 days. Four subscales (i.e., restraint, eating concern, shape concern, and weight concern) are used to evaluate frequency of disordered thoughts and behaviors. Item responses are scored on a 7-point Likert scale ranging from 0 (none of the time) to 6 (every time). EDE-Q subscale scores are calculated by determining the average of each subscale. An EDE-Q global score is calculated by adding the subscale scores and dividing by the total number of subscales. Higher scores indicate higher levels of disordered eating behaviors. Previous research suggests that EDE-Q global scores above 1.68 for men and 2.3 for women differentiate individuals with and without subclinical eating pathology (Mond et al., 2004; Schaefer et al., 2018). The EDE-Q item responses exhibit acceptable to excellent internal consistency (α = 0.73–0.93) and has been validated in a sample of college women (J. S. Rose et al., 2013). The EDE-Q total score demonstrates good test–retest reliability (r = 0.66–0.94; Berg et al., 2012) and is positively correlated with measures of eating concern (r = 0.68) and shape concern (r = 0.78; Mond et al., 2004), demonstrating criterion validity. Internal consistency for the EDE-Q in the present sample was excellent, with Cronbach’s alpha of 0.91 and McDonald’s omega of 0.95.

#### 2.3.4. Underreporting of Disordered Eating Behaviors Scale (UDEBS; Howard et al., 2022)

This validity scale was developed to detect attempts by respondents to present themselves in a favorable light with respect to their eating behaviors. Given that the DDEBS-12 asks participants to self-report denial, this measure was used to exclude participants who were less likely to respond honestly to the scale. The scale includes 10 true and false items describing undesirable eating habits that almost everyone engages in (e.g., “I sometimes eat too much”). The UDEBS was modeled after the Minnesota Multiphasic Personality Inventory (MMPI) Lie (L) scales (Ben-Porath & Tellegen, 2011). Total scores are calculated as the sum of all items endorsed in the socially desirable direction, ranging from 0 to 10, with higher scores representing more underreporting. Those who scored above 1 SD on the UDEBS were considered invalid and subsequently removed from analysis (Howard et al., 2022). The UDEBS was validated in a college sample using a simulation design wherein participants who were instructed to hide an eating disorder reported significantly higher UDEBS total scores than those instructed to fill out the questionnaire truthfully, demonstrating criterion validity (Howard et al., 2022).

#### 2.3.5. Distress Disclosure Index (DDI; Kahn & Hessling, 2001)

The DDI is a 12-item scale designed to measure the degree to which a person is comfortable talking with others about personally distressing information. Items are rated on a 5-point Likert scale, with responses ranging from 1 (strongly disagree) to 5 (strongly agree). Total scores range from 12 to 60, with higher scores indicating a higher willingness to disclose problems. DDI scores have shown stable test–retest reliabilities across 2- and 3-month periods of 0.80 and 0.81, respectively (Miller et al., 1983). The DDI has been validated with undergraduate women (Kahn et al., 2012). Internal consistency has been shown to be high across studies, ranging from 0.92 to 0.95 (Kahn et al., 2002, 2012). Internal consistency for the 12-item Distress Disclosure Index (DDI) in the present sample was excellent, with Cronbach’s alpha of 0.90 and McDonald’s omega of 0.95.

#### 2.3.6. Self-Concealment Scale (SCS; Larson & Chastain, 1990)

The SCS is a 10-item self-report inventory designed to measure a person’s general tendency to conceal personal information that is distressing. The SCS uses a 5-point Likert scale ranging from 1 (strongly disagree) to 5 (strongly agree). Total scores are calculated by summing all items, with higher scores indicating greater self-concealment. SCS scores are a reliable measure of self-concealment in college populations with test–retest and alpha estimates of 0.81 and 0.83, respectively (Larson et al., 2015). Cronbach’s alpha for the current sample was 0.90, and McDonald’s omega was 0.93.

#### 2.3.7. Eating Pathology Symptom Inventory—Restricting Subscale (EPSI; Forbush et al., 2013)

The restricting subscale of the EPSI is a 6-item measure designed to assess physical efforts to prevent or limit food consumption. The restricting subscale uses a 5-point Likert scale ranging from 0 (never) to 4 (very often). Total scores are calculated by summing all items, with higher scores indicating greater dietary restriction. There is evidence of test–retest reliability as well as convergent and discriminant validity in undergraduate samples (Forbush et al., 2013, 2014). Cronbach’s alpha for the current sample was 0.93, and McDonald’s omega was 0.91.

### 2.4. Data Analytic Plan

All models were conducted using Maximum Likelihood Robust (MLR) estimation to adjust for non-normality of the data. First, confirmatory factor analysis (CFA) was used to test whether the unidimensional factor solution of the original DDEBS-12 (Howard et al., 2020) was replicated in the present data. Model fit was evaluated based on the widely accepted goodness-of-fit indices specified by Hu and Bentler (1999): Root Mean Square Error of Approximation (RMSEA): ≤0.06; Comparative Fit Index (CFI): ≥0.95; Tucker–Lewis Index (TLI): ≥0.95; and Standardized Root Mean Square Residual (SRMR) ≤ 0.08. In the event that the original factor structure, which was established in a subclinical all-female-identifying college sample, did not fit the present data well, alternative models were estimated, using theory and measurement considerations as guides for model specification. Alternative models were not fully pre-specified but were theory-informed and tested following failure of the original model, consistent with an exploratory model refinement approach.

Second, a series of multi-group CFAs to test for measurement invariance were run across the following groups: gender identity, racial identity, and level of engagement in disordered eating. A series of nested models with increasing levels of equality constraints imposed across groups were used to test configural, metric, and scalar invariance. Evidence of invariance at any level precludes further invariance testing at the more stringent levels. Given the nested nature of the models, a chi-square difference test can be used to test the equality hypothesis. Because the models were estimated using MLR estimation, a Satorra–Bentler (SB) scaled chi-square difference test (Satorra & Bentler, 2010), which adjusts the chi-square statistics for non-normality, can be used to statistically compare models estimated using MLR. Although the SB scaled chi-square difference tests adjust for violations of normality, chi-square tests remain sensitive to sample size, and, thus, minor discrepancies can lead to false evidence of non-invariance (Brannick, 1995; Chen, 2007). Due to these and other drawbacks of chi-square difference tests, many researchers have opted for alternative criteria for comparing nested models, which are based on the relative changes in sample-size-independent fit indices. Specifically, changes in CFI and RMSEA greater than or equal to 0.010 signify meaningful differences in fit across the nested models, indicating non-invariance (Cheung & Rensvold, 2002; Chen, 2007). Thus, SB chi-square difference test statistics will be reported for transparency, but changes in CFI and RMSEA ≥ 0.01 were used to determine invariance.

## 3. Results

### 3.1. Examining the DDEBS Factor Structure in a College Sample

CFA was used to test the original factor structure of the DDEBS-12 using the 12 items published by Howard et al. (2020). In the original study, the residual variances for five items were allowed to correlate given the presence of theoretically related item content. Thus, the one-factor model was run allowing these residual variances to correlate, which resulted in inadequate model fit (χ^2^(44) = 721.45, *p* < 0.001; CFI = 0.918; TLI = 0.877; RMSEA = 0.068). To ensure that the lack of replicability of the original DDEBS-12 factor structure in the current sample was unrelated to the composition of the sample (i.e., a more generalized sample compared to the subclinical sample in the original paper; Howard et al., 2020), a CFA with subclinical women only was run, which also fit the data poorly (χ^2^(54) = 744.65, *p* < 0.001; CFI = 0.828; TLI = 0.789; RMSEA = 0.111). Therefore, a series of alternative models were estimated, using theory and measurement considerations as guides for model specification.

Although the DDEBS-12 items were not designed to assess denial of specific eating behaviors (e.g., denial of binge eating), the 12 items defined and validated by Howard et al. (2020) naturally split into two potential groupings: type of denial (verbal vs. behavioral) and type of eating behavior being denied (restrictive eating vs. overeating). These groupings align with retrospective survey data that suggest forms of denial can be split into verbal or behavioral (Vandereycken & Van Humbeeck, 2008) and theory that suggests restrictive eating and overeating often serve different functions, with restrictive eating related to a drive for thinness and overeating serving an emotion regulation function (Thompson & Stice, 2001; Lavender et al., 2015). Thus, a two-factor model accounting for the shared variance across items representing verbal denial (e.g., “Told people you have eaten when you have not eaten”) and items representing behavioral denial (e.g., “Eaten in secret”) was tested. This model demonstrated poor model fit (χ^2^(53) = 945.37, *p* < 0.001; CFI = 0.892; TLI = 0.865; RMSEA = 0.071).

Next, a two-factor model accounting for the shared variance across items representing denial of restriction (e.g., “Ate less food than you lead others to believe?”) and denial of overeating (e.g., “Ate more food than you lead others to believe?”) was tested, excluding three items that were ambiguously worded and could be interpreted either way (e.g., “Been dishonest about how much you ate”), and allowing the residual variances of the four items starting with “Told people” to correlate. This model fit the data well (χ^2^(20) = 151.98, *p* < 0.001; CFI = 0.975; TLI = 0.955; RMSEA = 0.045). However, the overeating factor specified in this model included only two indicators, which, according to DeVellis and Thorpe (2021), is not ideal given that subscales containing fewer than three items tend to have unstable factor loadings and are susceptible to random error. A one-factor model excluding the two overeating items was subsequently run. This model fit the data well (χ^2^(8) = 80.65, *p* < 0.001; CFI = 0.982; TLI = 0.953; RMSEA = 0.052). The revised DDEBS scale including items reflecting denial of restrictive eating behaviors and aptly named DDEBS-restriction, as well as their standardized factor loadings, is summarized in Table 2.

### 3.2. Invariance Across Groups

Measurement-invariance tests were subsequently conducted using multigroup CFA to determine whether the newly established factor structure of the DDEBS-restriction replicated across gender identity, racial identity, and level of engagement in disordered eating. Results are presented in Table 3. First, configural invariance was tested by analyzing the factor structure across the various groups without equality constraints. All configural models fit the data well, and all factor loadings were significant (*p* < 0.001) across all three models. This suggests that the structure of the DDEBS-restriction is invariant across participants identifying as men and women, Black and White, and across levels of engagement in disordered eating. Next, metric invariance was tested by placing equality constraints on the factor loadings across groups. Although the SB scaled χ^2^ difference test was significant (*p* = 0.016) when comparing the metric model for gender identity to the configural model, the change values of CFI (Δ = 0.003) and RMSEA (Δ = 0.004) were both smaller than 0.01, providing evidence to support metric invariance. The SB χ^2^ difference test was not significant, and changes in CFI and RMSEA were smaller than 0.01 in the racial identity and clinical models, providing evidence of metric invariance across racial identity and levels of disordered eating. Third, scalar invariance was tested by constraining the item intercepts to be equal across groups. The SB scaled χ^2^ difference tests were significant (*p* < 0.001) for gender identity and racial identity, but when comparing the scalar model to the metric model for both groups, the change values of CFI and RMSEA were smaller than 0.01, providing evidence that the means of the DDEBS-restriction construct did not statistically differ across gender identity or racial identity. In the model testing invariance across levels of disordered eating, the change value of RMSEA (Δ = 0.009) was smaller than 0.01, but the change value of CFI (Δ = 0.019) exceeded the suggested cutoff of 0.01, suggesting differences across groups at the scalar level. Additionally, the SB scaled χ^2^ difference test was significant (*p* < 0.001) when compared to the metric model. These findings suggest that the means of the DDEBS-restriction differ across non-clinical versus elevated disordered eating individuals.

### 3.3. Reliability and Validity

The seven-item DDEBS-restriction item responses possessed good internal consistency, with Cronbach’s alpha and McDonald’s omega both equal to 0.89. Convergent validity of the DDEBS-restriction total score was supported with a positive correlation with the Self-Concealment Scale (*r* = 0.35; *p* < 0.001) and a negative correlation with the Distress Disclosure Index (*r* = −0.13; *p* < 0.001). A moderate positive correlation between the DDEBS-restriction and the restriction subscale of the Eating Pathology Symptom Inventory indicated criterion validity (*r* = 0.48; *p* < 0.001). A full intercorrelation matrix among the primary study variables is provided in the Supplementary Materials.

## 4. Discussion

Previous research on disordered eating and related behaviors has primarily utilized samples of White women (Romano et al., 2020) despite an increased understanding that disordered eating can affect individuals across all racial and gender identities (Brown & Keel, 2023; Cheng et al., 2019; Halbeisen et al., 2022). Thus, there is a growing need to confirm whether measures of disordered eating and associated behaviors are appropriate to use with men and racial identities aside from White. The aim of the present study was to re-examine the factor structure of the DDEBS-12 and test measurement invariance of a revised DDEBS, the DDEBS-restriction, among different racial and gender identities. In addition, this study explored whether the DDEBS-restriction is appropriate for use in individuals who engage in various levels of disordered eating, including subthreshold levels. Lastly, the validity of the DDEBS-restriction was explored.

### 4.1. Factor Structure

Prior to exploring measurement invariance, a CFA was used to test whether the unidimensional factor solution of the DDEBS-12 (Howard et al., 2020) was replicated in the present data. The sample in the present study consisted of both men and women who engaged in various levels of disordered eating, including non-clinical levels. Model fit indices did not support the original factor solution in this expanded sample, and a series of CFAs were subsequently run by conceptually grouping items into subscales. A unidimensional model that included seven items assessing denial of dietary restriction, termed DDEBS-restriction, best fit the data. The DDEBS-restriction reflects a narrowing of the original construct of denial of disordered eating, focusing specifically on denial of restrictive behaviors rather than broader denial processes. Denial of restrictive eating behaviors represents a more psychometrically coherent subtype of denial than denial of disordered eating broadly. Denial processes related to overeating or other eating behaviors may be more heterogeneous and therefore less adequately represented by a single latent construct. Thus, the present findings may reflect not simply a refinement of the original DDEBS-12 but a narrowing toward a more specific and internally consistent form of denial associated with restrictive eating behaviors.

A scale that assesses denial of dietary restriction may also have greater theoretical relevance and utility given that denial of illness is most commonly studied and observed in individuals with symptoms of anorexia (e.g., Abbate-Daga et al., 2013; Vitousek et al., 1998). People who restrict food often lie about disordered eating behaviors because they do not want someone to intervene on their behaviors, especially if they view their dietary restriction as adaptive (Nordbø et al., 2006). For some individuals who engage in dietary restriction, their behaviors become a part of their identity: a phenomenon known as egosyntonicity (Vandereycken, 2006). Therefore, a tendency to “fake good” is portrayed in defense of one’s sense of self. Other individuals who restrict food may deny eating disorder symptoms because their disordered eating behaviors give them a sense of self-efficacy or achievement, and they hide their behaviors from others so they will not lose this sense of control, especially within societies that value thinness (Vitousek et al., 1998). Isolating denial of restriction adds value because it captures the form of denial most central to eating disorder presentations.

### 4.2. DDEBS-Restriction Invariance 

Measurement invariance tests using multigroup CFA suggested that the factor structure was invariant across women and men, Black and White participants, and non-clinical and, at least, subclinical participants in the present data. Given that male body ideals tend to focus more on attaining muscle mass (Waling, 2017), it would be plausible that items assessing denial of eating habits out of a desire to be thin may not be as relevant for men as they are for women. However, in our sample, the means of the DDEBS-restriction construct did not statistically differ across men and women, suggesting that denial of dietary restriction may be similarly applicable to men in non-clinical populations. Similarly, the means of the DDEBS-restriction construct did not statistically differ across Black and White participants. Although some literature suggests group differences in help-seeking and disclosure processes (e.g., Ward et al., 2013), these patterns are highly context-dependent and may not generalize to the concealment of eating behaviors specifically. Research on cultural variation in eating disorder presentation indicates that stigma and barriers to recognition and care may shape how disordered eating is experienced and disclosed across groups (e.g., Becker et al., 2010; Marques et al., 2011), with Black individuals generally less likely to disclose disordered eating behaviors. Thus, the absence of mean differences should be interpreted cautiously, as the DDEBS-restriction may capture concealment processes that are expressed similarly at the item level while still being embedded within distinct cultural contexts. Lastly, while the factor structure of the DDEBS-restriction replicated across levels of engagement in disordered eating, individuals who engaged in, at least, subclinical levels of disordered eating scored higher on average on the DDEBS-restriction. This result is expected given that higher engagement in disordered eating allows for more opportunity to deny such behavior, providing further evidence of criterion validity. Overall, configural and metric invariance were supported across all groups; scalar invariance was not supported across levels of disordered eating, indicating mean-level differences between non-clinical participants and participants who reported elevated levels of disordered eating.

### 4.3. Additional Psychometric Properties of the DDEBS-Restriction

Results suggested that scores on the DDEBS-restriction are both reliable and valid. The seven-item scale possessed good internal consistency, and there was evidence of criterion and convergent validity. The DDEBS-restriction total score was positively associated with a measure of dietary restriction, thus demonstrating criterion validity. This correlation was strong enough to support criterion validity but also indicated that the DDEBS-restriction measured a construct beyond disordered eating itself. One possibility is that the DDEBS-restriction captures processes of concealment or minimization that co-occur with restrictive behavior, which is consistent with work on secrecy, ego-syntonic beliefs, and illness maintenance in anorexia nervosa (Abbate-Daga et al., 2013; Gregertsen et al., 2017; Vandereycken, 2006), which may explain both the shared variance and the observed differentiation from direct symptom measures. Of note, the magnitude of this association is consistent with validity evidence observed for the original DDEBS-12 (Howard et al., 2020), which demonstrated a similarly sized correlation with disordered eating symptoms. The convergence suggests that the DDEBS-restriction retains the core validity properties of the full scale while providing a more targeted assessment of denial related to restrictive eating. Convergent validity was supported by a positive correlation with the Self-Concealment Scale and a negative correlation with the Distress Disclosure Index. These correlations were small to moderate but were expected to be so, as denial of disordered eating is different from a tendency to withhold or share information generally.

### 4.4. Strengths, Limitations, and Future Directions

This study utilized a large, racially diverse sample of men and women, and robust methodological approaches to re-examine the factor structure of the DDEBS-12 and determine whether the revised factor structure replicated across gender identity, racial identity, and level of engagement in disordered eating. However, these findings cannot be generalized to clinical or community populations, racial identities besides Black and White, and gender identities beyond female or male. Invariance analyses were limited to participants identifying as Black or White, and individuals identifying with other racial or ethnic groups were excluded due to sample size constraints; this should be considered when interpreting findings related to racial invariance. Additionally, the reduction from 12 to 7 items narrowed the breadth of the construct, limiting coverage of the full range of denial behaviors originally intended by the DDEBS. In particular, the exclusion of items reflecting denial of overeating restricts the scale’s ability to capture denial processes associated with binge eating or other forms of overconsumption.

An additional limitation concerns the exclusion of participants scoring high on the UDEBS, a measure designed to detect underreporting and socially desirable responding related to eating behaviors. Although this approach was intended to improve psychometric interpretability by reducing invalid responding, it also introduces a conceptual tension given that the construct under investigation involves denial of disordered eating behaviors. Thus, the resulting factor structure may reflect a psychometrically cleaner, but potentially narrower, representation of denial-related processes. In other words, the exclusion procedure may have disproportionately removed individuals highest on the tendencies the scale is intended to assess. Importantly, however, the overall pattern of factor analytic findings remained consistent when analyses were conducted with and without participants scoring high on the UDEBS.

Future research should continue to examine the factor structure and psychometric properties of the DDEBS-restriction in clinical and community populations, and other racial and gender identities. Although the revised factor structure demonstrated good fit in the present sample, it has not yet been cross-validated in an independent sample. The present study utilized a college sample, and denial may be particularly relevant to this population due to heightened stigma and social pressures during this time period (Duarte et al., 2015; Eisenberg et al., 2011). Therefore, whether the DDEBS-restriction is well suited for use among non-college and older populations is worthy of further investigation. Furthermore, although individuals who are not currently in treatment for an eating disorder but engage in disordered eating may be particularly likely to deny problematic behaviors to avoid treatment settings, denial may be just as, if not more, applicable to those diagnosed with an eating disorder. In fact, denial of disordered eating is often a defining feature of clinical eating disorders, especially anorexia (Abbate-Daga et al., 2013; Vitousek et al., 1998). The DDEBS-restriction might be useful as part of a clinical intake to determine whether and how often a client engages in denial of their symptoms. However, these potential applications should be considered preliminary, as the present findings are based on a non-clinical, cross-sectional sample, and further validation in clinical and community populations is needed before such uses can be supported. The DDEBS-restriction could additionally be useful in identifying those at risk for developing an eating disorder, but additional research is needed to determine whether the DDEBS-restriction can detect risk for clinically significant disordered eating better than other methods.

### 4.5. Conclusions

This study re-examined the factor structure of the DDEBS-12 in a college sample, which resulted in a revised seven-item scale assessing denial of dietary restriction, the DDEBS-restriction, whose scores demonstrated reliability and validity. The DDEBS-restriction demonstrated support for configural and metric invariance across men and women, Black and White participants, and individuals with non-clinical versus, at least, subclinical levels of disordered eating. These findings provide preliminary support for the use of the revised scale within these groups in an undergraduate sample, although additional research is needed to examine generalizability across more diverse populations. Future research should continue to examine the factor structure of the DDEBS-restriction in various populations. The DDEBS-restriction should be used to investigate how denial of dietary restriction might differ across various groups, ultimately informing prevention and intervention programs.

## Figures and Tables

**Table 1:** Descriptive Statistics.

Variable	*M* (*SD*)
Age	21.28 (6.79)
DDEBS-12 total	10.22 (19.86)
EDE-Q global	1.79 (1.38)
UDEBS total	1.09 (1.05)
DDI total	34.80 (5.76)
SCS total	26.81 (10.35)
EPSI-restricting	13.80 (5.32)
Variable	*N* (%)
Gender Identity	
Female	2537 (77.2%)
Male	748 (22.8%)
Racial Identity	
White or European American	1451 (55.0%)
Black or African American Sexual Orientation	1186 (45.0%)
Heterosexual or Mostly Heterosexual	2784 (84.4%)
Bisexual	257 (7.8%)
Homosexual or Mostly Homosexual	164 (4.9%)
Other or Prefer Not to Answer	95 (2.9%)
Student Classification	
Freshmen	1352 (41.0%)
Sophomore	630 (19.1%)
Junior	649 (19.7%)
Senior	640 (19.4%)
Graduate	29 (0.8%)
Level of Engagement in Disordered Eating	
Non-clinical	2074 (63.0%)
Subclinical	1217 (37.0%)

Note. DDEBS = Deliberate Denial of Disordered Eating Behaviors; EDE-Q = Eating Disorder Examination-Questionnaire; UDEBS = Underreporting of Disordered Eating Behaviors Scale; DDI = Distress Disclosure Index; SCS = Self-Concealment Scale; EPSI = Eating Pathology Symptom Inventory.

**Table 2:** Instructions: The following questions are concerned with the PAST MONTH. Please read each question and circle the appropriate number to the right. Please answer all the questions. Some of these questions may be difficult to answer or may feel uncomfortable.

Item	Estimate	Standard Error
Item #1: Told people you have eaten when you have not eaten?	0.686	0.018
Item #3: Told people you are not hungry when you are?	0.664	0.015
Item #6: Ate slowly in order to give the impression that you are eating more than you are?	0.779	0.015
Item #8: Deliberately hid food (e.g., in a napkin) in order to give the impression you ate more than you did?	0.696	0.021
Item #10: Ate less food than you led others to believe?	0.800	0.014
Item #11: Told people you have dietary restrictions (e.g., gluten free) in order to avoid eating certain foods?	0.530	0.027
Item #12: Told people you felt sick in order to avoid eating?	0.737	0.020

DDEBS-restriction Scale Instructions, Item Descriptions, and Standardized Factor Loadings. Note. All factor loadings are significant at *p* < 0.001.

**Table 3:** Invariance Metrics.

	χ^2^	CFI	TLI	RMSEA	ΔSB χ^2^	ΔCFI	ΔRMSEA
Configural Model							
Gender	78.22	0.984	0.948	0.049			
Racial Identity	77.64	0.981	0.950	0.054			
DE Engagement	79.23	0.980	0.947	0.049			
Metric Model					Metric against Configural		
Gender	93.91	0.981	0.965	0.045	15.580 **	0.003	0.004
Racial Identity	87.15	0.980	0.962	0.047	9.559	0.001	0.007
DE Engagement	88.76	0.979	0.959	0.043	9.334	0.001	0.006
Scalar Model					Scalar against Metric		
Gender	125.841	0.975	0.962	0.046	39.659 ***	0.006	0.001
Racial Identity	117.10	0.972	0.959	0.049	38.142 ***	0.008	0.002
DE Engagement	153.70	0.960	0.940	0.052	100.059 ***	0.019 ^✧^	0.009

Note. *** < 0.001 ** < 0.05. ^✧^ = exceeds the suggested change cutoff. DE engagement = non-clinical vs. self-reported subclinical or clinical disordered eating.

## Data Availability

Data, analysis code, and research materials are not available, but can be made available upon request.

## Note

1 Participants scoring more than 1 SD above the mean on the UDEBS reported significantly lower levels of disordered eating compared to retained participants, consistent with the measure’s intended purpose of detecting underreporting. No significant demographic differences were observed between excluded and retained participants. Additionally, the pattern of factor-analytic results remained consistent when analyses were conducted both with and without participants scoring high on the UDEBS.

## Supplementary Materials

The following supporting information can be downloaded at https://www.mdpi.com/article/10.3390/bs16060898/s1, Table S1: Two-factor model representing type of denial (verbal vs. behavioral); Table S2: Two-factor model representing type of eating behavior (restrictive eating vs. overeating).
