# Supplementary material for: Examining the Factor Structure and Subgroup Invariance of the Deliberate Denial of Disordered Eating Behaviors Scale
Source: Behav Sci (Basel). 2026 Jun 2;16(6):898. doi: 10.3390/bs16060898 (PMC13295254; doi:10.3390/bs16060898)
Supplement: Supplementary file 1 [file behavsci-16-00898-s001.zip › behavsci-4248095-supplementary.pdf]

Supplemental Materials

Table S1.
Two-factor model representing type of denial (verbal vs. behavioral)

Item	Verbal Denial		Behavioral Denial	
	Estimate	Standard Error	Estimate	Standard Error
1. Told people you have eaten when you have not eaten?	.758	.014		
2. Made up excuses to avoid events where you know food will be served?	.772	.015		
3. Told people you are not hungry when you are?	.768	.012		
4. Been dishonest about how much you ate?	.756	.015		
5. Eaten in secret?			.702	.018
6. Ate slowly in order to give the impression that you are eating more than you are?			.770	.014
7. Quickly cleared your plate in order to hide how much food you ate?			.757	.015
8. Deliberately hid food (e.g., in a napkin) in order to give the impression you ate more than you did?			.722	.019
9. Ate more food than you led others to believe?			.681	.019
10. Ate less food than you led others to believe?			.740	.016
11. Told people you have dietary restrictions (e.g., gluten free) in order to avoid eating certain foods?	.538	.026		
12. Told people you felt sick in order to avoid eating?	.756	.016		

Table S2.
Two-factor model representing type of eating behavior (restrictive eating vs. overeating)

Item	Restrictive Eating		Overeating	
	Estimate	Standard Error	Estimate	Standard Error
1. Told people you have eaten when you have not eaten?	.677	.016		
2. Made up excuses to avoid events where you know food will be served?				
3. Told people you are not hungry when you are?	.670	.014		
4. Been dishonest about how much you ate?				
5. Eaten in secret?			.793	.018
6. Ate slowly in order to give the impression that you are eating more than you are?	.779	.014		
7. Quickly cleared your plate in order to hide how much food you ate?				
8. Deliberately hid food (e.g., in a napkin) in order to give the impression you ate more than you did?	.715	.020		
9. Ate more food than you led others to believe?			.774	.020
10. Ate less food than you led others to believe?	.779	.014		
11. Told people you have dietary restrictions (e.g., gluten free) in order to avoid eating certain foods?	.547	.026		
12. Told people you felt sick in order to avoid eating?	.741	.018		

Note. Greyed out items were not modeled due to ambiguous wording that could be interpreted as either restrictive eating or overeating.
